# Monotonicity properties and bounds for the complete *p*-elliptic integrals

**DOI:** 10.1186/s13660-018-1828-2

**Published:** 2018-09-12

**Authors:** Ti-Ren Huang, Shen-Yang Tan, Xiao-Yan Ma, Yu-Ming Chu

**Affiliations:** 1 Department of Mathematics, Zhejiang Sci-Tech Universityu, Hangzhou, China; 2 Taizhou Institute of Science and Technology, Nanjing University of Science and Technology, Taizhou, China; 3 Department of Mathematics, Huzhou University, Huzhou, China

**Keywords:** 33C05, 33E05, 26D20, Complete elliptic integral, Complete *p*-elliptic integral, Gaussian hypergeometric function, Monotonicity, Convexity

## Abstract

We generalize several monotonicity and convexity properties as well as sharp inequalities for the complete elliptic integrals to the complete *p*-elliptic integrals.

## Introduction

Let $p>1$ and $0\leq\theta\leq 1$. Then the function $\sin_{p}^{-1}(\theta)$ and the number $\pi_{p}$ are defined by

1.1 $$ \sin_{p}^{-1}(\theta)= \int_{0}^{\theta}\frac{1}{(1-t^{p})^{1/p}}\,dt $$

and

1.2 $$ \frac{\pi_{p}}{2}=\sin_{p}^{-1}(1)= \int_{0}^{1}\frac{1}{(1-t^{p})^{1/p}}\,dt=\frac{\pi}{p\sin(\pi/p)}= \frac{1}{p} B(1/p,1-1/p), $$

respectively, where *B* is the classical beta function. The inverse function of $\sin_{p}^{-1}(\theta)$ defined on $[0, \pi_{p}/2]$ is said to be the generalized sine function and denoted by $\sin_{p}$. From (1.1) and (1.2) we clearly see that $\sin_{2}(\theta)=\sin(\theta)$ and $\pi_{2}=\pi$. The generalized sine function $\sin_{p}(\theta)$ and $\pi_{p}$ appeared in the eigenvalue problem of one-dimensional *p*-Laplacian

$$ -\bigl( \bigl\vert u' \bigr\vert ^{p-2}u' \bigr)'=\lambda \vert u \vert ^{p-2} u,\quad u(0)=u(1)=0. $$

Indeed, the eigenvalues are given by $\lambda_{n}=(p-1) (n\pi_{p})^{p}$ and the corresponding eigenfunction to $\lambda_{n}$ is $u(x)=\sin_{p}(n\pi_{n}x)$ for each $n=1, 2, 3,\ldots $ . In the same way one can define the generalized cosine and tangent functions and their inverse functions [1–3].

Let $x\in (-1,1)$, and *a*, *b* and *c* be the real numbers with $c\neq 0,-1,-2,\ldots $ . Then the Gaussian hypergeometric function $F(a, b; c; x)$ [4–11] is defined by

1.3 $$ F(a,b;c;x)=\sum_{n=0}^{\infty}\frac{(a,n)(b,n)}{(c,n)}\frac{x^{n}}{n!}, $$

where $(a,n)$ denotes the shifted factorial function $(a,n)=a(a+1)\cdots (a+n-1)$, $n=1,2,\ldots $ , and $(a,0)=1$ for $a\neq 0$. The well-known complete elliptic integrals $\mathcal{K}(r)$ and $\mathcal{E}(r)$ [12–15] of the first and second kinds are respectively defined by

$$ \mathcal{K}(r)=\frac{\pi}{2}F \biggl(\frac{1}{2},\frac{1}{2};1;r^{2} \biggr)= \int_{0}^{\pi/2}\frac{d\theta}{\sqrt{1-r^{2}\sin^{2}\theta}}= \int_{0}^{1}\frac{dt}{\sqrt{(1-t^{2})(1-r^{2}t^{2})}} $$

and

$$ \mathcal{E}(r)=\frac{\pi}{2}F \biggl(\frac{1}{2},- \frac{1}{2};1;r^{2} \biggr)= \int_{0}^{\pi/2}{\sqrt{1-r^{2} \sin^{2}\theta}}\,d\theta= \int_{0}^{1}{\sqrt{\frac{1-r^{2}t^{2}}{1-t^{2}}}}\,dt. $$

Let $p\in (1,\infty)$ and $r\in [0,1)$. Then the complete *p*-elliptic integrals $\mathcal{K}_{p}(r)$ and $\mathcal{E}_{p}(r)$ [16, 17] of the first and second kinds are respectively defined by

1.4 $$ \mathcal{K}_{p}(r)= \int_{0}^{\pi_{p}/2}\frac{d\theta}{(1-r^{p}\sin_{p}^{p}\theta)^{1-1/p}}= \int_{0}^{1}\frac{dt}{(1-t^{p})^{1/p}(1-r^{p}t^{p})^{1-1/p}} $$

and

1.5 $$ \mathcal{E}_{p}(r)= \int_{0}^{\pi_{p}/2}{\bigl(1-r^{p} \sin_{p}^{p}\theta\bigr)^{1/p}}\,d\theta= \int_{0}^{1}{ \biggl(\frac{1-r^{p}t^{p}}{1-t^{p}} \biggr)^{1/p}}\,dt. $$

From (1.4) and (1.5) we clearly see that the complete *p*-elliptic integrals $\mathcal{K}_{p}(r)$ and $\mathcal{E}_{p}(r)$ respectively reduce to the complete elliptic integrals $\mathcal{K}(r)$ and $\mathcal{E}(r)$ if $p=2$. Recently, the complete *p*-elliptic integrals $\mathcal{K}_{p}(r)$ and $\mathcal{E}_{p}(r)$ and their special cases $\mathcal{K}(r)$ and $\mathcal{E}(r)$ have attracted the attention of many mathematicians [18–30].

Takeuchi [31] generalized several well-known theorems for the complete elliptic integrals $\mathcal{K}(r)$ and $\mathcal{E}(r)$, such as Legendre’s formula, Gaussian’s $AGM$ approximation formulas for *π*, differential equations, and other similar results of the theory of complete elliptic integrals to the complete *p*-elliptic integrals $\mathcal{K}_{p}(r)$ and $\mathcal{E}_{p}(r)$, and proved that

1.6 $$\begin{aligned} &\mathcal{K}_{p}(r)=\frac{\pi_{p}}{2}F \biggl( \frac{1}{p},1-\frac{1}{p};1;r^{p} \biggr), \end{aligned}$$

1.7 $$\begin{aligned} &\mathcal{E}_{p}(r)=\frac{\pi_{p}}{2}F \biggl( \frac{1}{p},-\frac{1}{p};1;r^{p} \biggr). \end{aligned}$$

Anderson, Qiu, and Vamanamurthy [32] discussed the monotonicity and convexity properties of the function

$$ r\mapsto f(r)=\frac{\mathcal{E}(r)-r^{\prime2}\mathcal{K}(r)}{r^{2}}\cdot\frac{r^{\prime2}}{\mathcal{E'}(r)-r^{2}\mathcal{K'}(r)} $$

and proved that the double inequality

1.8 $$ \frac{\pi}{4}< f(r)< \frac{\pi }{4}+ \biggl( \frac{4}{\pi}-\frac{\pi}{4} \biggr)r $$

holds for all $r\in (0,1)$. Both inequalities given in (1.8) are sharp as $r\rightarrow 0$, while the second inequality is also sharp as $r\rightarrow 1$. Here and in what follows, we denote $r'=(1-r^{p})^{1/p}$, $\mathcal{K}_{p}'(r)=\mathcal{K}_{p}(r')$, and $\mathcal{E}_{p}'(r)=\mathcal{E}_{p}(r')$.

Alzer and Richards [33] proved that the function

$$ r\mapsto \Delta(r)=\frac{\mathcal{E}(r)-r^{\prime2}\mathcal{K}(r)}{r^{2}}-\frac{\mathcal{E'}(r)-r^{2}\mathcal{K'}(r)}{r^{\prime2}} $$

is strictly increasing and convex from $(0,1)$ onto $(\pi/4-1,1-\pi/4)$, and the double inequality

1.9 $$ \frac{\pi}{4}-1+\alpha r< \Delta(r)< \frac{\pi }{4}-1+\beta r $$

holds for all $r\in (0,1)$ with the best constant $\alpha=0$ and $\beta=2-\pi/2$.

Inequalities (1.8) and (1.9) have been generalized to the generalized elliptic integrals by Huang et al. in [34].

The main purpose of the article is to generalize inequalities (1.8) and (1.9) to the complete *p*-elliptic integrals. We discuss the monotonicity and convexity properties of the functions

1.10 $$\begin{aligned} &r\mapsto f_{p}(r)=\frac{\mathcal{E}_{p}(r)-r^{\prime p}\mathcal{K}_{p}(r)}{r^{p}}\cdot \frac{r^{\prime p}}{\mathcal{E'}_{p}(r)-r^{p}\mathcal{K'}_{p}(r)}, \end{aligned}$$

1.11 $$\begin{aligned} &r\mapsto g_{p}(r)=\frac{\mathcal{E}_{p}(r)-r^{\prime p}\mathcal{K}_{p}(r)}{r^{p}}- \frac{\mathcal{E'}_{p}(r)-r^{p}\mathcal{K'}_{p}(r)}{r^{\prime p}}, \end{aligned}$$

and present their corresponding sharp inequalities.

## Lemmas

In order to prove our main results, we need several formulas and lemmas, which we present in this section.

The following formulas for the hypergeometric function and complete *p*-elliptic integrals can be found in the literature [5, 1.20(10), (1.16), 1.19(4), (1.48)]], [18], and [35, Equation (26)]:

2.1 $$\begin{aligned} &F(a,b;a+b+1;x)=(1-x)F(a+1,b+1;a+b+1;x), \end{aligned}$$

2.2 $$\begin{aligned} &\frac{dF(a,b;c;x)}{dx}=\frac{ab}{c}F(a+1,b+1;c+1;x), \end{aligned}$$

2.3 $$\begin{aligned} &F(a,b;c;1)=\frac{\Gamma(c)\Gamma(c-a-b)}{\Gamma(c-a)\Gamma(c-b)} \quad (c>a+b), \end{aligned}$$

2.4 $$\begin{aligned} &F(a,b;c;x)\sim -\frac{\log(1-x)}{B(a,b)} \quad (x\rightarrow1, c=a+b), \end{aligned}$$

2.5 $$\begin{aligned} &(\sigma-\rho)F(\alpha,\rho;\sigma+1;z)=\sigma F(\alpha,\rho; \sigma;z)-\rho F(\alpha,\rho+1;\sigma+1;z), \end{aligned}$$

2.6 $$\begin{aligned} &\frac{d\mathcal {K}_{p}(r)}{dr}=\frac{\mathcal {E}_{p}(r)-r^{\prime p}\mathcal {K}_{a}(r)}{rr^{\prime p}}, \qquad \frac{d\mathcal {E}_{a}(r)}{dr}=\frac{\mathcal {E}_{p}(r)-{\mathcal {K}_{p}}(r)}{r}, \end{aligned}$$

where $\Gamma(x)$ is the classical gamma function.

### Lemma 2.1

*Let* $p \in(1,\infty)$. *Then the function*

2.7 $$ r\mapsto f_{p}^{1}(r)=\frac{\mathcal{E}_{p}(r)-r^{\prime p} \mathcal{K}_{p}(r)}{r^{p}} $$

*is strictly increasing and convex from* $(0, 1)$ *onto* $((p-1){\pi_{p}}/(2p), 1)$.

### Proof

It follows from (1.3), (1.6), and (1.7) that

$$\begin{aligned} &\mathcal{E}_{p}(r)-r^{\prime p} \mathcal{K}_{p}(r) \\ &\quad =\frac{\pi_{p}}{2} \biggl[F \biggl(\frac{1}{p},-\frac{1}{p}, \;1;r^{p} \biggr)-\bigl(1-r^{p}\bigr)F \biggl( \frac{1}{p},1-\frac{1}{p};1;r^{p} \biggr) \biggr] \\ &\quad =\frac{\pi_{p}}{2} \Biggl[\sum_{n=0}^{\infty}\frac{ (\frac{1}{p},n ) (-\frac{1}{p},n )}{(n!)^{2}}r^{pn}- \bigl(1-r^{p}\bigr)\sum _{n=0}^{\infty}\frac{ (\frac{1}{p},n ) (1-\frac{1}{p},n )}{(n!)^{2}}r^{pn} \Biggr] \\ &\quad =\frac{\pi_{p}}{2} \Biggl[\sum_{n=0}^{\infty}\frac{ (\frac{1}{p},n ) (-\frac{1}{p},n )- (\frac{1}{p},n ) (1-\frac{1}{p},n )}{(n!)^{2}}r^{pn}+ \sum_{n=0}^{\infty}\frac{ (\frac{1}{p},n ) (1-\frac{1}{p},n )}{(n!)^{2}}r^{p(n+1)} \Biggr] \\ &\quad =\frac{\pi_{p}}{2}\sum_{n=1}^{\infty}\frac{n^{2} (\frac{1}{p},n-1 ) (1-\frac{1}{p},n-1 ) -n (\frac{1}{p},n ) (1-\frac{1}{p},n-1 )}{(n!)^{2}}r^{pn} \\ &\quad =\frac{\pi_{p}}{2}\sum_{n=1}^{\infty}\frac{n (\frac{1}{p},n-1 ) (1-\frac{1}{p},n-1 ) (1-\frac{1}{p} )}{(n!)^{2}}r^{pn} \\ &\quad =\frac{\pi_{p} r^{p}}{2} \biggl(1-\frac{1}{p} \biggr)\sum _{n=0}^{\infty}\frac{1}{n+1} a_{n} r^{pn}, \end{aligned}$$

where $a_{n}=(1/p,n)(1-1/p,n)(n!)^{-2}$. Therefore,

2.8 $$ f_{p}^{1}(r)=\frac{\mathcal{E}_{p}(r)-r^{\prime p} \mathcal{K}_{p}(r)}{r^{p}}= \frac{\pi_{p}}{2} \biggl(1-\frac{1}{p} \biggr)\sum _{n=0}^{\infty}\frac{1}{n+1} a_{n} r^{pn} $$

and $f_{p}^{1}(r)$ is strictly increasing and convex on $(0, 1)$ due to $1-1/p>0$.

From (1.5), (1.6), (2.4), (2.7), and (2.8) we clearly see that

$$\begin{aligned} &\mathcal{E}_{p} \bigl(1^{-} \bigr)=1, \qquad \lim _{r\rightarrow 1^{-}}r^{\prime p} \mathcal{K}_{p}(r)=0, \\ &f_{p}^{1}\bigl(0^{+}\bigr)=\frac{(p-1){\pi_{p}}}{2p}, \qquad f_{p}^{1}\bigl(1^{-}\bigr)=1. \end{aligned}$$

 □

### Lemma 2.2

(see [18, Lemma 2.3])

*Let* $I\subset \mathbb{R}$ *be an interval and* $f, g: I\rightarrow (0,\infty)$ *be two positive real*-*valued functions*. *Then the product* *fg* *is convex on* *I* *if both* *f* *and* *g* *are convex and increasing* (*decreasing*) *on* *I*.

### Lemma 2.3

*Let* $p>1$. *Then the function*

2.9 $$\begin{aligned} r\mapsto J(r)=\frac{(1-r^{p}) (\mathcal{E}_{p}(r)-(1+r^{p})\mathcal{K}_{p}(r) )}{r^{2p-1}} \end{aligned}$$

*is strictly increasing from* $(0,1)$ *onto* $(-\infty,0)$.

### Proof

Let

2.10 $$ f_{1}(r)=\bigl(1-r^{p}\bigr) \biggl[F \biggl( \frac{1}{p},-\frac{1}{p};1;r^{p} \biggr)- \bigl(1+r^{p}\bigr)F \biggl(\frac{1}{p},1-\frac{1}{p};1;r^{p} \biggr) \biggr]. $$

Then it follows from (1.3), (1.6), (1.7), (2.9), and (2.10) that

2.11 $$\begin{aligned} &J(r)=\frac{\pi_{p}}{2r^{2p-1}}f_{1}(r), \end{aligned}$$

2.12 $$\begin{aligned} &f_{1}(r)=\bigl(1-r^{p}\bigr) \Biggl[\sum _{n=0}^{\infty}\frac{ (\frac{1}{p},n ) (-\frac{1}{p},n )}{(n!)^{2}}r^{pn}- \bigl(1+r^{p}\bigr)\sum_{n=0}^{\infty}\frac{ (\frac{1}{p},n ) (1-\frac{1}{p},n )}{(n!)^{2}}r^{pn} \Biggr] \\ &\hphantom{f_{1}(r)}=\bigl(1-r^{p}\bigr) \Biggl[\sum_{n=0}^{\infty}\frac{ (\frac{1}{p},n ) (-\frac{1}{p},n )- (\frac{1}{p},n ) (1-\frac{1}{p},n )}{(n!)^{2}}r^{pn}- \sum_{n=0}^{\infty}\frac{ (\frac{1}{p},n ) (1-\frac{1}{p},n )}{(n!)^{2}}r^{p(n+1)} \Biggr] \\ &\hphantom{f_{1}(r)}=\bigl(1-r^{p}\bigr)\sum_{n=1}^{\infty}\frac{-n^{2} (\frac{1}{p},n-1 ) (1-\frac{1}{p},n-1 ) -n (\frac{1}{p},n ) (1-\frac{1}{p},n-1 )}{(n!)^{2}}r^{pn} \\ &\hphantom{f_{1}(r)}=\bigl(1-r^{p}\bigr)\sum_{n=1}^{\infty}\frac{ (\frac{1}{p},n-1 ) (1-\frac{1}{p},n-1 ) ( (1-\frac{1}{p} )n-2n^{2} )}{(n!)^{2}}r^{pn} \\ &\hphantom{f_{1}(r)}=\sum_{n=1}^{\infty}\frac{ (\frac{1}{p},n-1 ) (1-\frac{1}{p},n-1 ) ( (1-\frac{1}{p} )n-2n^{2} )}{(n!)^{2}}r^{pn} \\ &\hphantom{f_{1}(r)=}{}-\sum_{n=2}^{\infty}\frac{ (\frac{1}{p},n-2 ) (1-\frac{1}{p},n-2 ) ( (1-\frac{1}{p} )(n-1)-2(n-1)^{2} )}{((n-1)!)^{2}}r^{pn} \\ &\hphantom{f_{1}(r)}= \biggl(-1-\frac{1}{p} \biggr)r^{p} \\ &\hphantom{f_{1}(r)=}{}+\sum _{n=2}^{\infty}\frac{ (\frac{1}{p},n-2 ) (1-\frac{1}{p},n-2 ) [2n (n+\frac{1}{p^{3}}-2 )+\frac{1}{p^{3}}-\frac{2}{p^{2}}-\frac{1}{p}+2 ]}{(n!)^{2}}r^{pn}. \end{aligned}$$

Equations (2.11) and (2.12) lead to

2.13 $$ \begin{aligned}[b] J(r)={}&\frac{\pi_{p}}{2} \Biggl\{ -\frac{1+p}{pr^{p-1}}\\ &{}+ \sum _{n=2}^{\infty}\frac{ (\frac{1}{p},n-2 ) (1-\frac{1}{p},n-2 ) [2n (n+\frac{1}{p^{3}}-2 ) +\frac{1}{p^{3}}-\frac{2}{p^{2}}-\frac{1}{p}+2 ]}{(n!)^{2}}r^{p(n-2)+1} \Biggr\} . \end{aligned} $$

It is easy to verify that

2.14 $$ 2n \biggl(n+\frac{1}{p^{3}}-2 \biggr)+\frac{1}{p^{3}}- \frac{2}{p^{2}}-\frac{1}{p}+2>0 $$

for $p>1$ and $n\geq 2$.

Therefore, the monotonicity of $J(r)$ on the interval $(0, 1)$ follows easily from (2.13) and (2.14).

From (2.3), (2.4), (2.10), (2.11), and (2.13) we clearly see that $J(0^{+})=-\infty$ and

$$\begin{aligned} &\lim_{r\rightarrow 1^{-}}F \biggl(\frac{1}{p}, -\frac{1}{p}; 1; r^{p} \biggr)=\frac{1}{\Gamma(1-1/p)\Gamma(1+1/p)}, \qquad \lim_{r\rightarrow 1^{-}} \bigl(1-r^{p} \bigr)F \biggl(\frac{1}{p}, 1-\frac{1}{p}; 1; r^{p} \biggr)=0, \\ &J\bigl(1^{-}\bigr)=0. \end{aligned}$$

 □

### Lemma 2.4

(see [5, Theorem 1.25])

*Let* $a, b\in \mathbb{R}$ *with* $a< b$, $f,g: [a,b]\rightarrow \mathbb{R}$ *be continuous on* $[a, b]$ *and be differentiable on* $(a, b)$ *such that* $g'(x) \neq 0$ *on* $(a, b)$. *If* $f'(x)/g'(x)$ *is increasing* (*decreasing*) *on* $(a, b)$, *then so are the functions*

$$ \frac{f(x)-f(a)}{g(x)-g(a)}, \quad\quad \frac{f(x)-f(b)}{g(x)-g(b)}. $$

*If* $f'(x)/g'(x)$ *is strictly monotone*, *then the monotonicity in the conclusion is also strict*.

## Main results

### Theorem 3.1

*Let* $p>1$ *and* $f_{p}(r)$ *be defined by* (1.10). *Then* $f_{p}(r)$ *is strictly increasing and convex from* $(0,1)$ *onto* $((p-1)\pi_{p}/(2p), 2p/[(p-1)\pi_{p}])$, *and the double inequality*

3.1 $$ \frac{(p-1) \pi_{p} }{2p}+\alpha r< f_{p}(r)< \frac{(p-1) \pi_{p} }{2p}+ \beta r $$

*holds for all* $r\in(0,1)$ *if and only if* $\alpha\leq 0$ *and* $\beta\geq 2p/[(p-1)\pi_{p}]-(p-1)\pi_{p}/(2p)$. *Moreover*, *both inequalities in* (3.1) *are sharp as* $r\rightarrow 0$, *while the second inequality is sharp as* $r\rightarrow 1$.

### Proof

Let $f^{1}_{p}(r)$ be defined by (2.7). Then $f_{p}(r)$ can be rewritten as

3.2 $$ f_{p}(r)=\frac{\mathcal{E}_{p}(r)-r^{\prime p}\mathcal{K}_{p}(r)}{r^{p}}\cdot\frac{r^{\prime p}}{\mathcal{E'}_{p}(r)-r^{p}\mathcal{K'}_{p}(r)}=f^{1}_{p}(r) \cdot \frac{1}{f^{1}_{p}(r')}. $$

From Lemma 2.1 we know that both the functions $f_{p}^{1}(r)$ and $1/f^{1}_{p}(r')$ are positive and strictly increasing on $(0,1)$, hence $f_{p}(r)$ is also strictly increasing on $(0,1)$.

Next, we prove that $1/f^{1}_{a}(r')$ is convex on $(0,1)$. It follows from (2.6) and (2.7) that

3.3 $$\begin{aligned} \frac{d}{dr} \biggl(\frac{1}{f_{p}^{1}(r)} \biggr)&= \frac{pr^{p-1}(\mathcal{E}_{p}(r)-r^{\prime p}\mathcal{K}_{p}(r))-(p-1)r^{2p-1}\mathcal{K}_{p}(r) }{(\mathcal{E}_{p}(r)-r^{\prime p}\mathcal{K}_{p}(r))^{2}} \\ &=\frac{p(\mathcal{E}_{p}(r)-r^{\prime p}\mathcal{K}_{p}(r))-(p-1)r^{p}\mathcal{K}_{p}(r) }{\frac{(\mathcal{E}_{p}(r)-r^{\prime p}\mathcal{K}_{p}(r))^{2}}{r^{p-1}}}=\frac{g_{1}(r)}{g_{2}(r)}, \end{aligned}$$

where

3.4 $$ \begin{aligned} &g_{1}(r)=p \bigl(\mathcal{E}_{p}(r)-r^{\prime p} \mathcal{K}_{p}(r) \bigr)+(1-p)r^{p}\mathcal{K}_{p}(r), \qquad g_{2}(r)=\frac{ (\mathcal{E}_{p}(r)-r^{\prime p}\mathcal{K}_{p}(r) )^{2}}{r^{p-1}}, \\ &\frac{g'_{1}(r)}{g'_{2}(r)}=\frac{r^{2p-1}}{(1-r^{p}) (\mathcal{E}_{p}(r)-(1+r^{p})\mathcal{K}_{p}(r) )}=\frac{1}{J(r)}, \end{aligned} $$

where $J(r)$ is defined by (2.9).

From (1.3), (1.6), (1.7), and Lemma 2.1 we clearly see that

3.5 $$ g_{1}\bigl(0^{+}\bigr)=g_{2} \bigl(0^{+}\bigr)=0. $$

Equations (3.3)–(3.5) and Lemmas 2.3 and 2.4 lead to the conclusion that the function $\frac{d}{dr} (1/f_{p}^{1}(r) )$ is strictly decreasing on $(0, 1)$, which implies that the function $\frac{d}{dr} (1/f_{p}^{1}(r') )$ is strictly increasing on $(0, 1)$ and $1/f^{1}_{a}(r')$ is convex on $(0,1)$.

Therefore, $f_{p}(r)$ is convex on $(0, 1)$ follows from Lemmas 2.1 and 2.2 together with (3.2) and the convexity of $1/f^{1}_{a}(r')$.

The limit values

3.6 $$ f_{p}\bigl(0^{+}\bigr)=\frac{(p-1)\pi_{p}}{2p}, \qquad f_{p}\bigl(1^{-}\bigr)=\frac{2p}{(p-1)\pi_{p}} $$

follow easily from Lemma 2.1 and (3.2).

It follows from (2.8) and (3.2) that

3.7 $$\begin{aligned} &\lim_{r\rightarrow 0^{+}}\frac{df_{p}(r)}{dr} \\ &\quad =\lim_{r\rightarrow 0^{+}}\frac{\sum_{n=0}^{\infty}\frac{a_{n}}{n+1}(1-r^{p})^{n}\sum_{n=1}^{\infty}\frac{pna_{n}}{n+1}r^{pn-1} +r^{p-1}\sum_{n=0}^{\infty}\frac{a_{n}}{n+1}r^{pn}\sum_{n=1}^{\infty}\frac{pna_{n}}{n+1}(1-r^{p})^{n-1}}{ [\sum_{n=0}^{\infty}\frac{a_{n}}{n+1}(1-r^{p})^{n} ]^{2}} \\ &\quad=0. \end{aligned}$$

Therefore, inequality (3.1) holds for all $r\in(0,1)$ if and only if $\alpha\leq 0$, and $\beta\geq 2p/[(p-1)\pi_{p}]-((p-1)\pi_{p}/(2p)$ follows easily from (3.6) and (3.7) together with the monotonicity and convexity of $f_{p}(r)$ on $(0, 1)$. From (3.6) we clearly see that both inequalities in (3.1) are sharp as $r\rightarrow 0$ and the second inequality is sharp as $r\rightarrow 1$. □

### Theorem 3.2

*Let* $p\geq 2$ *and* $g_{p}(r)$ *be defined in* (1.11). *Then* $g_{p}(r)$ *is strictly increasing and convex from* $(0,1)$ *onto* $((p-1)\pi_{p}/(2p)-1, 1-(p-1)\pi_{p}/(2p))$, *and the double inequality*

3.8 $$ \frac{(p-1)\pi_{p}}{2p}-1+\alpha r< g_{p}(r)< \frac{(p-1)\pi_{p}}{2p}-1+\beta r $$

*holds for all* $r \in (0, 1)$ *if and only if* $\alpha\leq 0$ *and* $\beta\geq 2-(p-1)\pi_{p}/p$. *Moreover*, *both inequalities in* (3.8) *are sharp as* $r\rightarrow 0$, *while the second inequality is sharp as* $r\rightarrow 1$.

### Proof

Let

$$ M_{p}(r)=\frac{\pi_{p}}{2r^{p}} \biggl[F \biggl(\frac{1}{p},- \frac{1}{p};1;r^{p} \biggr)-r^{\prime p}F \biggl(\frac{1}{p},1-\frac{1}{p};1;r^{p} \biggr) \biggr]. $$

Then from (1.3), (1.6), (1.7), and (1.11) we get

3.9 $$\begin{aligned} & M_{p}(r)=\frac{\pi_{p}(p-1)}{2p}F \biggl(1- \frac{1}{p},\frac{1}{p};2;r^{p} \biggr), \end{aligned}$$

3.10 $$\begin{aligned} &\begin{aligned}[b] g_{p}(r)&=M_{p}(r)-M_{p} \bigl(r'\bigr)\\ &=\frac{\pi_{p}(p-1)}{2p} \biggl[F \biggl(1- \frac{1}{p},\frac{1}{p};2;r^{p} \biggr)-F \biggl(1- \frac{1}{p},\frac{1}{p};2;1-r^{p} \biggr) \biggr]. \end{aligned} \end{aligned}$$

It follows from (2.1), (2.2), (2.5), and (3.10) that

3.11 $$\begin{aligned} &\frac{dg_{p}(r)}{dr}=\frac{(p-1)^{2}\pi_{p}}{4p^{2}}r^{p-1} \biggl[F \biggl(2- \frac{1}{p}, 1+\frac{1}{p};3;r^{p} \biggr)+F \biggl(2- \frac{1}{p},1+\frac{1}{p};3;1-r^{p} \biggr) \biggr], \\ &\frac{dg_{p}(r)}{dr}|_{r=0}=0, \end{aligned}$$

3.12 $$\begin{aligned} &\begin{aligned}[b] \frac{d^{2}g_{p}(r)}{dr^{2}}={}&\frac{(p-1)^{3}\pi_{p}}{4p^{2}}r^{p-2} \biggl[F \biggl(2- \frac{1}{p},1+\frac{1}{p};3;r^{p} \biggr)+F \biggl(2- \frac{1}{p},1+\frac{1}{p};3;1-r^{p} \biggr) \\ &{}+\frac{\pi_{p}(p-1)^{2}(1+p)(2p-1)}{12p^{3}}r^{2p-2} \\ &{}\times\biggl(F \biggl(3-\frac{1}{p},2+ \frac{1}{p};4;r^{p} \biggr) -F \biggl(3-\frac{1}{p},2+ \frac{1}{p};4;1-r^{p} \biggr) \biggr) \biggr], \end{aligned} \end{aligned}$$

3.13 $$\begin{aligned} &F \biggl(3-\frac{1}{p},2+\frac{1}{p};4;1-r^{p} \biggr)=\frac{1}{r^{p}}F \biggl(2-\frac{1}{p},1+\frac{1}{p};4;1-r^{p} \biggr), \end{aligned}$$

3.14 $$\begin{aligned} &\begin{aligned}[b] &\biggl(2-\frac{1}{p} \biggr)F \biggl(2- \frac{1}{p},1+\frac{1}{p};4;1-r^{p} \biggr) \\ &\quad =3F \biggl(2-\frac{1}{p},1+\frac{1}{p};3;1-r^{p} \biggr) - \biggl(1+\frac{1}{p} \biggr)F \biggl(2-\frac{1}{p},2+ \frac{1}{p};4;1-r^{p} \biggr). \end{aligned} \end{aligned}$$

Equations (1.3) and (3.12)–(3.14) lead to

3.15 $$\begin{aligned} &\frac{4p^{2}}{(p-1)^{2}\pi_{p}}r^{p-2}g''_{p}(r) \\ &\quad = (p-1)F \biggl(2-\frac{1}{p},1+\frac{1}{p};3;r^{p} \biggr) +(p-1)F \biggl(2-\frac{1}{p},1+\frac{1}{p};3;1-r^{p} \biggr) \\ &\qquad {}+\frac{(2p-1)(p+1)}{3p}r^{p}F \biggl(3-\frac{1}{p},2+ \frac{1}{p};4;r^{p} \biggr) \\ &\qquad {}-r^{p}\frac{(2p-1)(p+1)}{3p}F \biggl(3-\frac{1}{p},2+\frac{1}{p};4;1-r^{p} \biggr) \\ &\quad = (p-1)F \biggl(2-\frac{1}{p},1+\frac{1}{p};3;r^{p} \biggr)+(p-1)F \biggl(2-\frac{1}{p},1+\frac{1}{p};3;1-r^{p} \biggr) \\ &\qquad {}+\frac{(2p-1)(p+1)}{3p}r^{p}F \biggl(3-\frac{1}{p},2+ \frac{1}{p};4;r^{p} \biggr) \\ &\qquad {} -\frac{(2p-1)(p+1)}{3p}F \biggl(2- \frac{1}{p},1+\frac{1}{p};4;1-r^{p} \biggr) \\ &\quad = (p-1)F \biggl(2-\frac{1}{p},1+\frac{1}{p};3;r^{p} \biggr)+\frac{(2p-1)(p+1)}{3p}r^{p}F \biggl(3-\frac{1}{p},2+ \frac{1}{p};4;r^{p} \biggr) \\ &\qquad {}+(p-1)F \biggl(2-\frac{1}{p},1+\frac{1}{p};3;1-r^{p} \biggr)-(2p-1)F \biggl(2-\frac{1}{p},1+\frac{1}{p};3;1-r^{p} \biggr) \\ &\qquad {}+\frac{(2p-1)^{2}}{3p}F \biggl(3-\frac{1}{p},1+\frac{1}{p};4;1-r^{p} \biggr) \\ &\quad = (p-1)F \biggl(2-\frac{1}{p},1+\frac{1}{p};3;r^{p} \biggr)+\frac{(2p-1)(p+1)}{3p}r^{p}F \biggl(3-\frac{1}{p},2+ \frac{1}{p};4;r^{p} \biggr) \\ &\qquad {}-pF \biggl(2-\frac{1}{p},1+\frac{1}{p};3;1-r^{p} \biggr)+\frac{(2p-1)^{2}}{3p}F \biggl(1+\frac{1}{p},3-\frac{1}{p};4;1-r^{p} \biggr) \\ &\quad > p-1-pF \biggl(2-\frac{1}{p},1+\frac{1}{p};3;1-r^{p} \biggr)+\frac{(2p-1)^{2}}{3p}F \biggl(1+\frac{1}{p},3-\frac{1}{p};4;1-r^{p} \biggr) \\ &\quad = p-1-p\sum_{n=0}^{\infty}\frac{(1+\frac{1}{p},n)(2-\frac{1}{p},n)}{(3,n)} \frac{(1-r^{p})^{n}}{n!} \\ &\qquad {}+\frac{(2p-1)^{2}}{3p}\sum_{n=0}^{\infty} \frac{(1+\frac{1}{p},n)(3-\frac{1}{p},n)}{(4,n)}\frac{(1-r^{p})^{n}}{n!} \\ &\quad = \sum_{n=0}^{\infty}\frac{(1+\frac{1}{p},n)(2-\frac{1}{p},n)}{(3,n+1)} \biggl[(p-1)n+p+\frac{1}{p}-4 \biggr]\frac{(1-r^{p})^{n}}{n!} \\ &\quad \geq \sum_{n=0}^{1}\frac{(1+\frac{1}{p},n)(2-\frac{1}{p},n)}{(3,n+1)} \biggl[(p-1)n+p+\frac{1}{p}-4 \biggr]\frac{(1-r^{p})^{n}}{n!} \\ &\quad = \frac{20p^{4}-36p^{3}-p^{2}+6p-1}{12p^{3}}>0 \end{aligned}$$

for $p\geq 2$.

Therefore, the monotonicity and convexity for $g_{p}(r)$ on the interval $(0, 1)$ follow from (3.11) and (3.15).

It follows from (1.2), (1.3), (2.3), (3.9), and (3.10) that

3.16 $$ \begin{aligned} &M_{p}\bigl(0^{+}\bigr)=\frac{(p-1)\pi_{p}}{2p}, \qquad M_{p}\bigl(1^{-}\bigr)=1, \\ &g_{p}\bigl(0^{+}\bigr)=\frac{(p-1)\pi_{p}}{2p}-1, \qquad g_{p}\bigl(1^{-}\bigr)=1-\frac{(p-1)\pi_{p}}{2p}. \end{aligned} $$

Therefore, the desired results in Theorem 3.2 follow easily from (3.11) and (3.16) together with the monotonicity and convexity of $g_{p}(r)$ on the interval $(0, 1)$. □

### Remark 3.3

Let $p=2$. Then we clearly see that inequalities (3.1) and (3.8) reduce to inequalities (1.8) and (1.9), respectively.

### Corollary 3.4

*Let* $p\geq 2$, $g_{p}(r)$ *be defined by* (1.11) *and*

3.17 $$ L_{p}(x,y)=g_{p}(xy)-g_{p}(x)-g_{p}(y). $$

*Then the double inequality*

$$ \frac{(p-1)\pi_{p}}{2p}-1< L_{p}(x,y)< 1-\frac{(p-1)\pi_{p}}{2p} $$

*holds for all* $x,y\in (0,1)$.

### Proof

It follows from (3.17) and the proof of Theorem 3.2 that

3.18 $$\begin{aligned} &\frac{\partial}{\partial x}L_{p}(x,y)=yg'_{p}(xy)-g'_{p}(x), \end{aligned}$$

3.19 $$\begin{aligned} &\frac{\partial^{2}}{\partial x\partial y}L_{p}(x,y)=g'_{p}(xy)+xyg''_{p}(xy)>0 \end{aligned}$$

for all $x, y\in (0, 1)$.

From (3.17)–(3.19) we get

3.20 $$ \begin{aligned} &\frac{\partial}{\partial x}L_{p}(x,y)< \frac{\partial}{\partial x}L_{p}(x,y)|_{y=1}=0, \\ &{-}g_{p}(1)=L_{p}(1,y)< L_{p}(x,y)< L_{p}(0,y)=-g_{p}(y). \end{aligned} $$

Therefore, Corollary 3.4 follows easily from Theorem 3.2 and (3.20). □

## Methods

The main purpose of the article is to generalize inequalities (1.8) and (1.9) for the complete elliptic integrals to the complete *p*-elliptic integrals. To achieve this goal we discuss the monotonicity and convexity properties for the functions given by (1.10) and (1.11) by use of the analytical properties of the Gaussian hypergeometric function and the well-known monotone form of l’Hôpital’s rule given in [5, Theorem 1.25].

## Results and discussion

In the article, we present the monotonicity and convexity properties and provide the sharp bounds for the functions

$$ r\mapsto f_{p}(r)=\frac{\mathcal{E}_{p}(r)-r^{\prime p}\mathcal{K}_{p}(r)}{r^{p}}\cdot\frac{r^{\prime p}}{\mathcal{E'}_{p}(r)-r^{p}\mathcal{K'}_{p}(r)} $$

and

$$ r\mapsto g_{p}(r)=\frac{\mathcal{E}_{p}(r)-r^{\prime p}\mathcal{K}_{p}(r)}{r^{p}}-\frac{\mathcal{E'}_{p}(r)-r^{p}\mathcal{K'}_{p}(r)}{r^{\prime p}} $$

on the interval $(0, 1)$.

The obtained results are the generalization of the well-known results on the classical complete elliptic integrals given in [32, 33].

## Conclusion

In this paper, we generalize the monotonicity, convexity, and bounds for the functions involving the complete elliptic integrals to the complete *p*-elliptic integrals. The given idea may stimulate further research in the theory of generalized elliptic integrals.
